# High Thickness Tolerance in All‐Polymer‐Based Organic Photovoltaics Enables Efficient and Stable In‐Door Operation

**DOI:** 10.1002/advs.202408181

**Published:** 2024-09-20

**Authors:** Lei Zhang, Seonjeong Lee, Song Yi Park, Oskar J. Sandberg, Emily J. Yang, Paul Meredith, Yun‐Hi Kim, Ji‐Seon Kim

**Affiliations:** ^1^ Department of Physics and Centre for Processable Electronics Imperial College London Prince Consort Road London SW7 2AZ UK; ^2^ Department of Chemistry and Research Institute of Molecular Alchemy (RIMA) Gyeongsang National University Jinju Gyeongnam 660‐701 South Korea; ^3^ Physics, Faculty of Science and Engineering Åbo Akademi University Henrikinkatu 2 Turku 20500 Finland; ^4^ Sustainable Advanced Materials (Sêr SAM) Group Centre for Integrative Semiconductor Materials and Department of Physics Swansea University Singleton Park Swansea SA2 8PP UK; ^5^ Present address: Department of Physics Pukyong National University Busan 48513 Republic of Korea

**Keywords:** indoor photovoltaics, organic solar cells, polymer/polymer blends, space‐charge effect, thickness tolerance

## Abstract

Organic photovoltaics (OPVs) have great potential to drive low‐power consumption electronic devices under indoor light due to their highly tunable optoelectronic properties. Thick devices (>300 nm photo‐active junctions) are desirable to maximize photocurrent and to manufacture large‐scale modules via solution‐processing. However, thick devices usually suffer from severe charge recombination, deteriorating device performances. Herein, the study demonstrates excellent thickness tolerance of all‐polymer‐based PVs for efficient and stable indoor applications. Under indoor light, device performance is less dependent on photoactive layer thickness, exhibiting the best maximum power output in thick devices (34.7 µW cm^−2^ in 320–475 nm devices). Thick devices also exhibit much better photostability compared with thin devices. Such high thickness tolerance of all‐polymer‐based PV devices under indoor operation is attributed to strongly suppressed space‐charge effects, leading to reduced bimolecular recombination losses in thick devices. The unbalanced charge carrier mobilities are identified as the main cause for significant space‐charge effects, which is confirmed by drift‐diffusion simulations. This work suggests that all‐polymer‐based PVs, even with unbalanced mobilities, are highly desirable for thick, efficient, and stable devices for indoor applications.

## Graphical abstract

The excellent thickness tolerance of all‐polymer‐based bulk heterojunction organic photovoltaics is demonstrated for highly efficient and stable indoor applications. Thicker devices exhibit significantly suppressed space‐charge effects, reducing undesirable bimolecular recombination losses under indoor light conditions. This work provides a potential way to manufacture large‐scale modules by using a thick photoactive layer via solution processing.

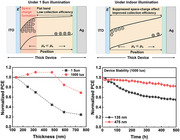

## 1 Introduction

Organic photovoltaics (OPVs) are a promising energy production technology due to their unique properties, such as flexibility, light weight, semi‐transparency, and compatibility with solution‐processing.^[^ ^1^, ^2^, ^3^ ^]^ Recently, OPVs have achieved remarkable progress thanks to the development of various non‐fullerene acceptors.^[^ ^4^ ^]^ Although power conversion efficiencies (PCEs) of OPVs currently exceed 20% under 1 Sun condition,^[^ ^5^ ^]^ they still lag behind their inorganic counterparts, such as silicon, GaAs, and hybrid organic‐inorganic perovskite solar cells.^[^ ^6^ ^]^ Therefore, it is important to explore other niche applications that are more suitable for OPVs and can take full advantage of their unique properties. Indoor application is one of those examples where OPVs are strongly competitive.^[^ ^7^, ^8^, ^9^ ^]^

Indoor photovoltaics can convert indoor light into electrical power to drive low‐power consumption electronic devices. Commonly used indoor lights, such as fluorescent lamps and light‐emitting diodes (LEDs), have emission spectra ranging from 300 to 800 nm, which is much narrower than the standard solar spectrum.^[^ ^9^ ^]^ Crystalline silicon solar cells show relatively lower PCEs under indoor light, due to mismatch between their absorption spectra with the LED emission spectra.^[^ ^10^ ^]^ On the contrary, optical band gaps of organic photoactive materials can be easily tuned by proper molecular design, enabling spectral overlap between their absorption and the emission of visible‐range wavelengths. Moreover, a high open‐circuit voltage (*V*_OC_) can be achieved even under indoor light condition by suitable donor and acceptor combinations or by controlling the energy levels of organic semiconductors. Therefore, OPVs are promising candidates for indoor applications compared to the inorganic counterparts due to the facile tunability of optoelectronic properties of organic semiconductor materials.^[^ ^11^, ^12^ ^]^

The intensity of indoor lights is typically less than 1 mW cm^−2^, which is more than two orders of magnitude lower than that of 1 Sun condition.^[^ ^13^ ^]^ Therefore, obtaining the maximum possible photocurrent is crucial to achieve highly efficient indoor OPVs. A feasible strategy to maximize photocurrent is to introduce thick photoactive layers, which not only facilitate enhanced light absorption but also minimize leakage currents and mitigate the risk of shunt reduction.^[^ ^14^ ^]^ Additionally, a thick photoactive layer of more than 300 nm is normally required for large‐area solution‐processed OPV module fabrication.^[^ ^15^, ^16^ ^]^ In general, optimum device performance under 1 Sun condition can be achieved with a relatively thin (≈100 nm) photoactive layer. Device performance is found to decrease with thicker photoactive layers mainly due to low and unbalanced charge carrier mobilities leading to poor charge transport and extraction properties.^[^ ^17^, ^18^ ^]^ Hence, achieving high efficiencies with the photoactive layer thickness of over 300 nm is one of the key challenges in OPV technology.^[^ ^19^, ^20^ ^]^ In particular, a space‐charge region is known to be formed at one of the electrodes if charge carrier mobilities are not balanced.^[^ ^21^, ^22^ ^]^ This space‐charge region can screen the internal electric field which may inhibit charge carrier extraction leading to severe recombination losses. In addition, significant charge trapping and unfavorable blend morphology can also limit device performance of thick OPVs.^[^ ^23^, ^24^ ^]^ Nonetheless, an improvement in fill factor (FF) has been observed for low‐light conditions, due to suppressed recombination losses induced by low photo‐generated charge carrier density.^[^ ^7^, ^25^ ^]^ This suggests that thick photoactive layer OPVs have great potential for efficient indoor light harvesting.

Yin et al. reported 200‐nm thick devices based on P1:PC71BM showing a PCE of 18.43% under 300 lux illumination.^[^ ^26^ ^]^ With the same thickness, they obtained a higher PCE of over 21% in P3TEA:FTTB‐PDI4‐based devices under illumination from 170 to 1650 lux.^[^ ^27^ ^]^ Shin et al. studied the effect of active layer thickness using PPDT2FBT:PC71BM.^[^ ^14^ ^]^ The optimal PCE is achieved with a thickness of 280 nm under 1 Sun and a thickness of 390 nm under LED illumination. The higher thickness tolerance under indoor light is explained by the ratio of series and shunt resistance, pointing to the importance of high shunt resistance in thick devices for efficient indoor operation. An ultra‐thick device based on PBDB‐T:N2200 for indoor applications has been reported by Wang et al., showing a PCE of 16.13% under 1000 lux even with a thickness of 1000 nm.^[^ ^28^ ^]^ The excellent device performance is realized by adding 5% polystyrene, which was attributed to effectively reduce trap states. All these promising results support the idea that thick active layers are suitable for indoor operation of OPVs; however, thick device architectures have still only received little attention in indoor photovoltaic research. Additionally, the difference in photovoltaic behaviors under 1 Sun and indoor light including the operational stability, has not yet been fully investigated or explained in detail.

In this study, we employed the polymer donor PBDB‐T and the polymer acceptor P(NDI2OD‐Se‐Th 0.8) for all‐polymer blends. The NDI‐based acceptor has achieved 8.3% efficiency under 1 Sun illumination with PBDB‐T, outperforming N2200.^[^ ^29^ ^]^ P(NDI2OD‐Se‐Th 0.8) has similar absorption to N2200 but with a slightly larger optical bandgap, making it promising for indoor applications. We demonstrate that all‐polymer OPVs exhibit excellent thickness tolerance under indoor light conditions. Under 1 Sun illumination, device performance declines with increased thickness due to space‐charge accumulation, which is induced by unbalanced charge transport and results in increased bimolecular recombination losses. In contrast, under indoor light (1000 lux), the device performance is less sensitive to thickness, with 320 nm thick devices achieving a maximum power output (*P*_max_) of 34.71 µW cm⁻^2^. This improvement is attributed to significantly reduced space‐charge effects under indoor lighting, resulting from the lower charge carrier density. Additionally, thick devices show better photostability compared to thin devices. These findings highlight that all‐polymer OPVs, despite unbalanced charge mobilities, are well‐suited for efficient and stable indoor applications.

## 2 Results and Discussion

### 2.1 Device Performances Under 1 Sun and Indoor Light

**Figure 1:**
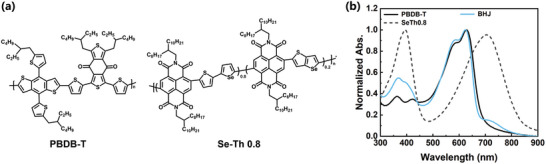
a) Molecular structure of PBDB‐T and P(NDI2OD‐Se‐Th 0.8). b) Normalized absorption spectra of neat and blend films.

**Figure 2:**
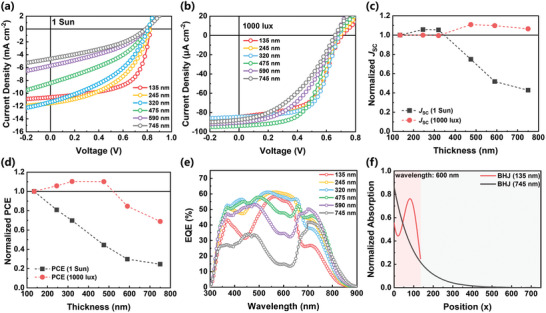
Representative *J*–*V* characteristics of various‐thickness all‐polymer solar cells measured under a) 1 Sun and b) 1000 lux condition. Normalized c) *J*_SC_ and d) PCE as a function of thickness measured under 1 Sun and 1000 lux conditions. e) EQE spectra of various‐thickness devices. f) Simulated absorption profiles of 135 and 745 nm devices at 600‐nm wavelength.

PBDB‐T and P(NDI2OD‐Se‐Th 0.8) are used as a donor and an acceptor, respectively, due to their superior spectral overlap between their absorption and the indoor LED emission spectrum (**Figure** **1**). The acceptor absorption diminishes after blending, a phenomenon also observed in other NDI‐based polymers.^[^ ^30^, ^31^ ^]^ This reduced contribution from the acceptor in the blend can be attributed to the blend ratio used (2:1, wt.%) and the inherently lower absorption coefficient of P(NDI2OD‐Se‐Th 0.8).^[^ ^29^ ^]^ Devices were fabricated with an inverted structure, with photoactive layer thickness varying from 135 to 745 nm (See Experimental Section for more details). As the thickness increases, the roughness of the active layer increases gradually without obvious morphological changes (Figure S1, Supporting Information). The devices were measured under two different light conditions: 1 Sun (AM 1.5G) using a solar simulator and indoor lights using a white LED. The emission spectrum of the white LED is shown in Figure S2 (Supporting Information). Here, *P*_max_ is used to evaluate device performance under LED illumination. Representative current density–voltage (*J*–*V*) characteristics for the devices, measured under 1 Sun and indoor LED (1000 lux) illumination are shown in **Figure** **2**. Corresponding photovoltaic parameters are summarised in **Table** **1**. Under 1 Sun illumination, the thinnest devices (135 nm) exhibit the highest FF of 0.60 and a relatively high short‐circuit current density (*J*_SC_) of 10.55 mA cm^−2^, resulting in the optimal PCE of 5.20%. As the photoactive layer thickness increases to 245 nm, the *J*_SC_ is slightly enhanced and reaches its maximum value of 11.41 mA cm^−2^, possibly due to enhanced absorption in thicker films. Despite the *J*_SC_ improvement, the FF drops to 0.47, leading to a reduced PCE of 4.40%. With a further thickness increase, *J*_SC_ values start to decrease sharply down to 4.60 mA cm^−2^ in thickest (745 nm) devices leading to the lowest PCE of 1.34%. The significant reduction in *J*_SC_ and FF in the thicker devices is likely related to the severe charge recombination which will be discussed in detail in following section.

**Table 1:** Summary of photovoltaic parameters under 1 Sun and 1000 lux conditions. (All parameters are averaged from over 8 devices).

Light intensity	Thickness [nm]	*J*_SC_ [mA cm^−2^]	*V*_OC_ [V]	FF	PCE [%]
1 Sun	135	10.55 ± 0.27	0.822 ± 0.006	0.600 ± 0.009	5.20 ± 0.13
	245	11.41 ± 0.23	0.816 ± 0.005	0.473 ± 0.018	4.40 ± 0.15
	320	11.39 ± 0.32	0.808 ± 0.005	0.411 ± 0.012	3.79 ± 0.21
	475	8.02 ± 0.59	0.801 ± 0.003	0.375 ± 0.001	2.41 ± 0.18
	590	5.55 ± 0.35	0.793 ± 0.002	0.367 ± 0.001	1.61 ± 0.10
	745	4.60 ± 0.46	0.782 ± 0.006	0.370 ± 0.011	1.34 ± 0.19

Light intensity	Thickness [nm]	*J*_SC_ [µA cm^−2^]	*V*_OC_ [V]	FF	*P*_max_ [µW cm^−2^]
1000 lux	135	83.01 ± 2.41	0.703 ± 0.006	0.539 ± 0.020	31.46 ± 1.88
	245	82.97 ± 2.84	0.689 ± 0.003	0.582 ± 0.016	33.28 ± 2.00
	320	82.58 ± 2.56	0.687 ± 0.003	0.611 ± 0.015	34.71 ± 1.63
	475	92.25 ± 3.19	0.673 ± 0.002	0.558 ± 0.008	34.65 ± 1.10
	590	91.17 ± 2.24	0.664 ± 0.002	0.440 ± 0.016	26.64 ± 1.30
	745	88.42 ± 4.06	0.656 ± 0.004	0.374 ± 0.024	21.67 ± 1.41

Very different photovoltaic behavior is observed under the low light (1000 lux) excitation conditions. Compared to the 1 Sun, the FF of thicker devices (245‐590 nm) exhibit a significant enhancement. Benefiting from the best FF of 0.61, the 320 nm devices show the highest *P*_max_ of 34.71 µW cm^−2^. Interestingly, the highest *J*_SC_ of 92.25 µA cm^−2^ is achieved in thicker devices (475 nm) and further increase in the thickness does not reduce this high *J*_SC_ significantly. These results indicate that it is possible to maximize the *J*_SC_ by applying a thick polymer/polymer blend photoactive layer, as it not only allows more photons to be absorbed, but also minimizes the leakage current (Figure S3, Supporting Information). Due to the maintained high *J*_SC_ obtained for the ultrathick photoactive layer, the 745 nm devices can still yield a *P*_max_ of 21.67 µW cm^−2^.

For a clear comparison, the thickness dependence of normalized photovoltaic parameters under 1 Sun and 1000 lux conditions are plotted, with *J*_SC_ and PCE shown in Figure 2c,d, and *V*_OC_ and FF shown in Figure S4 (Supporting Information). Consistent with other reported thick OPVs,^[^ ^21^, ^32^, ^33^ ^]^ the *V*_OC_ only shows a negligible variation with different photoactive layer thicknesses and has a minor impact on the efficiency. The slight reduction of *V*_OC_ in thick devices may originate from the increased recombination loss.^[^ ^21^, ^34^ ^]^ In terms of FF, however, as the photoactive layer thickness increases to 500 nm, enhanced FFs are observed compared to the thinnest devices under indoor light condition, unlike those of 1 Sun measurement which show gradual decrease of FF. This indicates charge transport and extraction properties are not limited by thicker photoactive layer under low light conditions. *J*_SC_ trends are clearly different depending on photoactive layer thickness as well as illumination conditions. Under 1 Sun, an abrupt reduction of *J*_SC_ is observed when the thickness is >400 nm, whereas *J*_SC_ measured under indoor light is maintained (or even higher) with increase of thickness. As a result, the PCE is sensitive to thickness variation and decreases drastically in thick devices under the 1 Sun condition. In contrast, the device efficiency shows a higher thickness tolerance under the 1000 lux condition with thick devices (245–475 nm) yielding better efficiencies than thin devices.

External quantum efficiency (EQE) spectra were measured to evaluate the charge collection efficiency, as shown in Figure 2e. Notably, a large discrepancy between the measured *J*_SC_ (*J*_SC, Measured_) from *J*–*V* curves and calculated *J*_SC_ from EQE spectra (*J*_SC, EQE_) is observed (Figure S5, Supporting Information). The detailed values of *J*_SC, EQE_ are summarised in Table S1 (Supporting Information). The *J*_SC, EQE_ values increase with thickness, reaching a maximum at 320 nm, and even the value of the 590 nm device is larger than that of the 135 nm device. The *J*_SC, EQE_ values show higher thickness tolerance similar to the *J*_SC_ measured under 1000 lux. When the active layer thickness increases above 200 nm, the calculated *J*_SC, EQE_ is much larger than the *J*_SC, Measured_. The possible reason for this discrepancy is the low intensity of the incident light of EQE measurements, placing devices in low‐light operation scenarios.^[^ ^35^, ^36^ ^]^ Similar *J*_SC_ values in thin devices arise from low recombination losses under both 1 Sun and low light conditions. In thick devices, suppressed recombination losses during low‐light EQE measurements lead to an overestimation of *J*_SC_.^[^ ^37^ ^]^ This observation aligns with their enhanced FF and sustained high *J*_SC_ under the 1000 lux condition as discussed above. Interestingly, spectral changes of EQE spectra are also observed with a significant reduction ≈600 nm wavelengths in thicker devices.

To confirm whether this effect arises from wavelength‐dependent absorption, absorption profiles were simulated. According to the simulated absorption profiles of 135 and 745 nm devices (Figure 2f), light with wavelength of 600 nm is mostly absorbed near the (semi‐transparent) cathode (ZnO) in the thickest devices. Therefore, photogenerated holes in the 745 nm thick devices can recombine more easily, since they have to travel a longer distance to the anode, resulting in limited charge extraction in this spectral range (≈600 nm). As the absorption profiles at the other wavelengths are relatively uniform within the active layer regardless of photoactive layer thicknesses (Figure S6, Supporting Information), the EQE shape change is only obvious at ≈600 nm. This significant EQE thickness dependence on a particular excitation wavelength originates from strong absorption property of the photoactive blend layer at 600 nm.

### 2.2 Charge Recombination and Transport

To evaluate the charge recombination loss, light‐intensity dependent measurements were performed. The slope extracted from the dependence of *V*_OC_ upon light intensities provides information about the extent of trap‐assisted recombination.^[^ ^38^ ^]^ As shown in Figure S7 (Supporting Information), the slopes are close to *kT*/*q* for thick devices under both solar simulator and LED illumination, suggesting negligible trap‐assisted recombination. To obtain further details about charge trapping, ambient photoemission spectroscopy (APS) and surface photovoltage (SPV) measurements were performed.^[^ ^11^, ^39^, ^40^, ^41^ ^]^ As shown in Figure S8a (Supporting Information), the thick films show a much smaller integrated area below the band edge, suggesting a lower density of shallow trap states.^[^ ^40^ ^]^ Moreover, when the light intensity is reduced to 1000 lux, a smaller drop in SPV magnitude is measured in thick films compared to thin films, suggesting less trap‐assisted recombination loss in thick films (Figure S8b, Supporting Information).^[^ ^11^, ^39^, ^40^, ^41^, ^42^ ^]^ Thus, it can be concluded that compared with thin devices, the dominant origin that limits the efficiency of thick devices is bimolecular recombination rather than trap‐assisted recombination.

**Figure 3:**
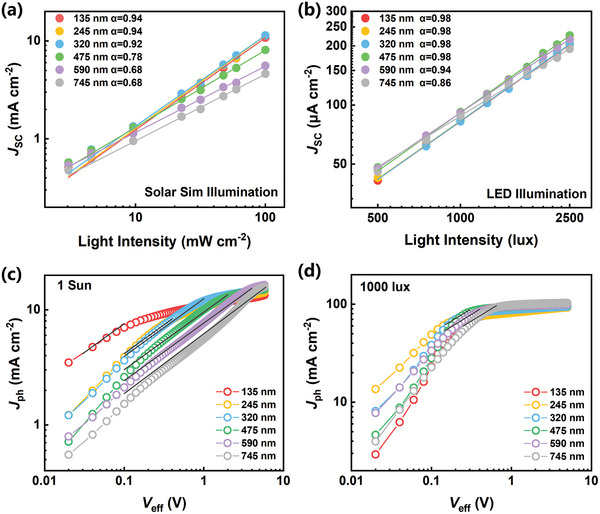
Light‐intensity dependence of *J*_SC_ measured using a) solar simulator and b) indoor light (1000 lux). *J*_ph_ versus *V*_eff_ characteristics measured under c) 1 Sun and d) 1000 lux conditions. The solid lines represent the square‐root dependence of the *J*_ph_ on *V*_eff_.

The relationship between *J*_SC_ and light intensity was investigated to further assess the degree of bimolecular recombination. The *J*_SC_ is expected to follow a power law relationship with light intensity (*I*) (*J*_SC_∝*I* ^α^). As α approaches unity, the influence of bimolecular recombination weakens.^[^ ^43^ ^]^ As shown in **Figure** **3**a, the *α* value is fitted to be 0.94 for thinnest devices under 1 Sun illumination, which indicates relatively efficient charge extraction. As the thickness increases, the *α* value drops significantly, implying thicker devices suffer from severe bimolecular recombination. This could explain the abrupt reduction in device performances of thick devices under 1 Sun conditions. Notably, all devices show increased *α* values under indoor LED illumination (Figure 3b), indicating suppressed bimolecular recombination. Even for the devices with a thickness up to 475 nm, a high *α* value of 0.98 is maintained. These results are consistent with the high FF and *J*_SC_ observed in thick devices under the 1000 lux condition.

**Figure 4:**
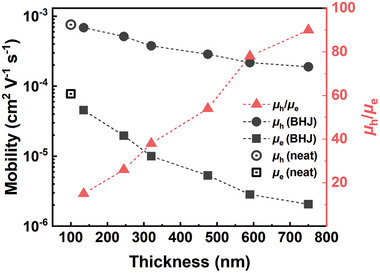
Hole/electron mobility ratio (*µ*_h_/*µ*_e_) and mobilities of neat and BHJ films as a function of thickness.

Since bimolecular recombination is related to the charge transport properties, hole and electron mobilities were measured using the space‐charge limited current (SCLC) method. **Figure** **4** summarizes the SCLC electron and hole mobilities of neat donor, neat acceptor, and various‐thickness BHJ blend devices. Detailed values are summarised in Table S2 (Supporting Information) and *J–* *V* curves of corresponding single‐carrier devices are shown in Figures S9 and S10 (Supporting Information). Compared with neat films, the SCLC hole and electron mobilities both decrease in blend films. As the blend film thickness increases, both hole and electron mobilities are gradually reduced. With a much stronger reduction in electron mobility, the larger difference in hole and electron mobilities is observed in thicker devices. Such unbalanced hole and electron mobilities in thicker devices may lead to space‐charge formation, especially in thick devices, which may be the origin of strong bimolecular recombination.

To prove the presence of a space‐charge region, effective photocurrent (*J*_ph_) as a function of effective voltage (*V*_eff_) was measured under 1 Sun and 1000 lux conditions. Here, *J*_ph_ is defined as *J*_ph_ = *J*_L_ – *J*_D_, where *J*_L_ and *J*_D_ are experimentally measured current densities under illumination and dark conditions, respectively. *V*_eff_ is defined as *V*_eff_ = *V*_0_ – *V*, where *V*_0_ is the voltage at which *J*_ph_ = 0 and *V* is the applied voltage. If a device is limited by space‐charge effect, *J*_ph_ is expected to show a square‐root dependence on *V*_eff_.^[^ ^17^, ^44^, ^45^ ^]^ Under 1 Sun conditions (Figure 3c), the thinnest devices show a small square‐root region. As the thickness increases, the square‐root region gets larger and extends to a higher voltage region. For devices with a thickness above 300 nm, even if *V*_eff_ reaches 0.8 V (short circuit condition), *J*_ph_ still lies within the square‐root region rather than the saturation region. This indicates that charges in thick devices are not extracted efficiently even by the internal electric field at short circuit condition, which is consistent with the inferior device performances of thick devices measured under 1 Sun illumination. However, the square‐root region is not observed in the thinnest devices under the 1000 lux condition (Figure 3d), indicating that charge extraction is not limited by space‐charge effects. For thicker devices, although a square‐root region may still be discerned, it is shifted toward lower voltages and its width gets significantly smaller, showing weaker space‐charge formation. This result agrees well with the suppressed bimolecular recombination in all devices under low‐light conditions.

In general, a square‐root dependence may also be obtained if *J*_ph_ is limited by trap‐assisted recombination (with constant carrier lifetime).^[^ ^46^ ^]^ To further confirm that *J*_ph_ is limited by space charge induced by unbalanced mobilities, a series of *J*_ph_ versus *V*_eff_ curves under different light intensities were characterized with the solar simulator (Figure S11, Supporting Information) and LED (Figure S12, Supporting Information) illumination. If *J*_ph_ is limited by space‐charge effect, it follows a 0.75 power dependence on light intensity.^[^ ^44^ ^]^ Moreover, saturation voltage (*V*_sat_) extracted from the crossover point between the square‐root region and the saturation region is expected to scale with a 0.5 power dependence on light intensity.^[^ ^44^ ^]^ The *J*_ph_ at *V*_eff_ = 0.2 V in the square‐root region and *V*_sat_ extracted from Figures S11 and S12 (Supporting Information) are plotted in Figure S13 (Supporting Information) as a function of light intensity.

Under solar simulator illumination, the fitted slopes of the 135 nm devices suggest they may be limited by trap‐assisted recombination.^[^ ^46^ ^]^ While the ≈0.75 power dependence of *J*_ph_ and the ≈0.5 power dependence of *V*_sat_ on light intensities confirm the presence of a space‐charge region in the thicker devices (>135 nm). When the light source is changed to indoor LED, only devices with a thickness above 320 nm show a slope of ≈0.75 fitted from the light dependent *J*_ph_. As expected, their *V*_sat_ shows a square‐root dependence, which gives a further indication of space‐charge formation in thick devices (>320 nm). These results confirm that under 1 Sun illumination, only the thinnest devices are free of space‐charge effects, while under low‐light conditions devices with a thickness of up to 320 nm are not limited by the space‐charge formation.

### 2.3 Simulation Validation for Space‐Charge Effects

**Figure 5:**
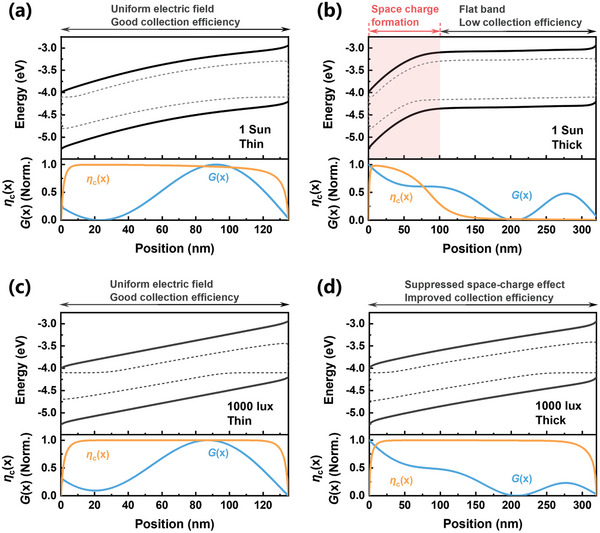
Simulated energy‐level diagrams and corresponding collection efficiency (*η*_c_(x)) and normalized generation rate (*G*(x)) plots of a,c) the thin (135 nm) device under (a) 1 Sun and (c) 1000 lux conditions at short circuit compared with b,d) the thick (320 nm) device.

In order to gain further insight into the space‐charge effect, we simulated band diagrams, absorption profiles, and charge collection profiles for active layer thicknesses of thin (135 nm) and thick (320 nm) under 1 Sun and 1000 lux conditions. In the 135 nm device, even with unbalanced mobilities (*µ*_h_/*µ*_e_ = 15), energy bands show approximately linear gradients without space‐charge region under both 1 Sun and low‐light conditions (**Figure** **5**a,c, respectively). In these thinner devices, a uniform and sufficiently high internal electric field is observed throughout the active layer, which can extract charges sufficiently. In the thicker device (320 nm), there is an obvious band bending observed under 1 Sun illumination (Figure 5b). Due to significantly lower electron mobilities in thicker devices as measured (*µ*_h ≫_ *µ*_e_), the electrons will accumulate at the cathode side resulting in the formation of a space‐charge region. The large photo‐generated electron density within this space‐charge region can screen the internal electric field in the rest of the active layer resulting in a flat‐band region where the charge carrier extraction is inefficient, inevitably leading to considerable photocurrent losses.

However, under 1000 lux illumination, the space‐charge region diminishes completely in 320 nm devices, resulting in efficient charge extraction across the active layer (Figure 5d). This is because the photo‐generated charge carrier density of electrons at this intensity is not large enough to screen the electric field in these devices. Even for ultrathick devices (590 nm), space‐charge formation is less significant for indoor light conditions, compared to those formed under 1 Sun condition (Figure S14, Supporting Information). Therefore, we conclude that the suppressed space‐charge effect is the key, which leads to excellent thickness tolerance of overall device performance under indoor light condition. Together with simulation and experimental results, we confirm that all polymer‐based BHJ blend systems, even with extremely unbalanced hole and electron mobilities can still be viable candidates to achieve thick and efficient OPVs for indoor application.

### 2.4 Stability

**Figure 6:**
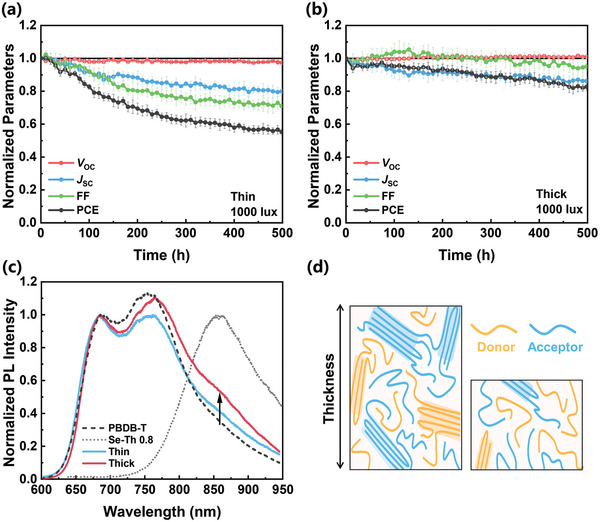
Normalized photovoltaic parameters (average values from 8 devices) decay of a) thin (135 nm) and b) thick (475 nm) devices under the 1000 lux condition. c) Normalized PL spectra of neat PBDB‐T and Se‐Th 0.8 films and thin and thick blend films. d) Schematic illustration of thin and thick film morphologies.

To further rationalize the use of thick photoactive layers and its benefit for practical indoor OPV applications, we tested the photostability of devices with different thicknesses (135 vs 475 nm). Photostability testing was conducted using a nitrogen‐filled chamber and measured under continuous white LED illumination with 1000 lux intensity. Importantly, there is clear difference between thin and thick devices in terms of operational photostability, even at very low light intensity conditions. For both thin and thick devices, the *V*_OC_ values do not exhibit noticeable changes. However, thin devices show PCE reduction by ≈40% after 500 h with respect to the initial PCE, mainly due to gradual decrease of *J*_SC_ and FF (**Figure** **6**a). In contrast, thicker devices show better photostability maintaining over 80% of the initial PCE value after 500 h, mainly due to smaller decrease of *J*_SC_ and FF compared to thin devices (Figure 6b).

To identify the origin of the better photostability in thicker devices, the photoluminescence (PL) spectra of neat and blend films were measured. Despite similar initial surface morphologies measured by atomic force microscopy (AFM) (Figure S1, Supporting Information), fresh blend films exhibit different features in PL spectra, indicating different hidden nanoscale morphologies. By comparing the initial PL intensity of thick and thin devices, as shown in Figure S15 (Supporting Information), it is evident that the thick devices exhibit higher intensity, indicating a reduced donor‐acceptor interface area.^[^ ^47^ ^]^ PL spectra of both thin and thick films show two peaks at 685 nm (0‐0 transition) and 755 nm (0‐1 transition) corresponding to donor emission,^[^ ^48^ ^]^ and a peak at 860 nm corresponding to acceptor emission (Figure 6c). The intensity ratio of 𝐼_0‐0_/𝐼_0‐1_ can serve as an indicator of the degree of aggregation. A lower ratio signifies a higher degree of aggregation.^[^ ^49^ ^]^ Compared to thin films, thick films show a lower 𝐼_0‐0_ intensity, resulting in a smaller ratio, which indicates more donor aggregation in thick films. Moreover, the peak ≈860 nm, originating from the acceptor, is stronger in thick films, further suggesting increased acceptor aggregation. This finding indicates less intermixing between donor and acceptor polymers in thick films, as evidenced by the greater aggregation of both polymers (Figure 6d; Figure S16, Supporting Information).

To gain further insight into the degradation process, the in situ PL was measured by using a laser excitation to accelerate degradation.^[^ ^50^, ^51^ ^]^ The corresponding spectra of neat and blend films at varied excitation times are shown in Figures S17 and S18 (Supporting Information). In general, there is a gradual decrease in PL intensity in both thin and thick blend films. In addition, both films show changes in the relative intensities of the donor emission peaks (685 and 755 nm) without any changes in the acceptor emission peak (860 nm) in the normalized PL spectra. The relative intensity of the high‐energy peak (685 nm) drops significantly, indicating more aggregation of donor polymers formed during photo‐aging. This is consistent with the larger domains and roughness observed in the AFM height images of aged films (Figure S19, Supporting Information). The initial rapid change in the relative intensity of the high‐energy peak can be attributed to the exceptionally high‐power intensity (≈300 W cm^−2^) of the lasers used for device degradation, which significantly accelerates the degradation process. For thin films such high‐energy peak (685 nm) intensity drop occurs continuously with prolonged photoexcitation indicating unstable blend morphology (Figure S18c, Supporting Information). In contrast, for thick films the shape of PL spectra remains almost unchanged after the rapid initial change, indicating a more stable overall active layer morphology (Figure S18d, Supporting Information).

Optimized blend morphology in organic solar cells typically resides in a thermally activated metastable state, rendering it susceptible to aging. Over time, thermodynamic relaxation of the mixed domains may occur, driving the system toward a thermodynamic equilibrium state and resulting in larger phase separation during degradation.^[^ ^47^, ^52^ ^]^ Enhancing acceptor aggregation through polymer doping has been reported to improve blend morphological stability under thermal stress.^[^ ^47^ ^]^ In this study, thick‐film devices exhibit greater phase segregation with increased donor and acceptor aggregation, leading to a relatively more thermodynamically stable structure. The in situ PL data suggests that this stability mitigates further morphological changes induced by prolonged photoexcitation, thereby enhancing long‐term photostability. Consequently, a thick active layer not only maximizes efficiency but also improves stability under indoor light conditions.

## 3 Conclusion

In conclusion, we have demonstrated excellent thickness tolerance of all‐polymer‐based photovoltaic devices for efficient and stable indoor applications. We find that bimolecular recombination is the dominant factor deteriorating the device performances of the all‐polymer OPVs with thicker active layers, which originates from the formation of a space‐charge region induced by unbalanced hole and electron mobilities. Under 1 Sun, the device performances, especially the *J*_SC_ and FF, degrade sharply upon thickness increase due to the prominent space‐charge effect in thick devices. However, a high thickness tolerance is observed under 1000 lux, with optimal efficiency achieved in much thicker devices (320–475 nm). This is enabled by suppressed bimolecular recombination loss with weaker space‐charge formation, which is verified by both experiment and simulation data. Our work also demonstrates the advantage of a thick active layer, even with unbalanced charge transport, in indoor applications, as it can yield the highest *J*_SC_ under low‐light conditions. In addition, thick devices exhibit better photostability upon continuous illumination. We suggest that a thick active layer using an all‐polymer blend system with high and balanced mobilities is promising for achieving high‐performing devices under indoor lights with superior efficiency and stability.

## 4 Experimental Section

### Material

PBDB‐T polymer was purchased from Solarmer. The trimethyl(5‐(5‐(trimethylstannyl)selenophen‐2‐yl)thiophen‐2‐yl)stannane, selenopheno[3,2‐b]thiophene‐2,5‐diylbis(trimethylstannane) (SeTh), 4,9‐dibromo‐2,7‐bis(2‐octyldodecyl)benzo[lmn][3,8]phenanthroline‐1,3,6,8(2H,7H)‐tetraone, and P(NDI2OD‐Se‐Th 0.8) was prepared by reported method. The *M*_n_ and PDI of P(NDI2OD‐Se‐Th 0.8) were 54.8 and 1.96, respectively.

### General

The *M*_n_ and *Ð* of the P(NDI2OD‐Se‐Th 0.8) were determined by gel permeation chromatography measurements with a Waters 1515 instrument equipped with a refractive index detector, in the condition of chloroform eluent at room temperature calibrated with polystyrene standards. The UV–vis absorbance data was obtained using a Shimadzu UV‐2550 UV‐visible spectrophotometer. SPV and APS were measured by an APS04 Air Photoemission system (KP Technology) using a 2 mm gold tip in ambient conditions. AFM height images were obtained using a Park NX‐10 AFM microscope in noncontact mode. In situ PL was measured using a Renishaw inVia Raman microscope with a 50× objective in a backscattering configuration. A 514‐nm laser excitation was used with a power density of ≈300 W cm^−2^. The PL was corrected for differing absorption at 514 nm.

### Device Fabrication Procedures and Characterization

All the devices were fabricated with an inverted structure of ITO/ZnO/active layer/MoO_3_/Ag. The pre‐patterned ITO substrates were cleaned in an ultrasonic bath with sequence of deionized water, acetone, and 2‐propanol for 15 min, then dried in an oven at 80 °C overnight. The substrates were treated with oxygen plasma for 3 min before ZnO coating. The ZnO solution prepared using the sol‐gel method was filtered and spin‐coated at 4000 rpm on ITO substrates, then the substrates were thermally annealed on a hotplate at 190 °C for 20 min. PBDB‐T:Se‐Th 0.8 (2:1 w/w blend ratio) blend solutions were dissolved in chlorobenzene and stirred at 100 °C for 2 h. By controlling concentrations and spin rates, thickness was controlled to 135 ± 5, 245 ± 1, 320 ± 4, 475 ± 5, 590 ± 5, and 745 ± 13 nm. Then the substrates were thermally annealed at 130 °C for 10 min in a N_2_ glovebox. After thermal annealing, MoO_3_ (10 nm) and Ag (100 nm) were deposited by thermal evaporation in a high vacuum chamber (≈10^−6^ Torr). The device area was 0.06 cm^2^. Despite optimization efforts, the device performance under 1 Sun falls below literature‐reported values.^[^ ^29^ ^]^ This discrepancy may stem from the fabrication in ambient air, in contrast to literature devices prepared in a glovebox.^[^ ^53^ ^]^

The devices were not encapsulated. They were stored in a nitrogen‐filled chamber and measured in air. The 1 Sun measurement was conducted using a solar simulator (100 mW cm^−2^). White LED light source (1000 lux) was used for indoor light measurement. A digital luxmeter was used for light intensity calibration of the white LED. It is stressed that these LED‐based measurements were not meant to align with an accepted standard, but merely to provide a controlled comparative measurement with a source indicative of indoor light. The *J*–*V* characteristics were measured with a Keithley 2400 source measurement unit. EQE was measured using an EQE system of a tungsten halogen lamp coupled with a grating spectrometer (CS260‐RG‐4‐MT‐D).

For SCLC measurements, electron‐only devices were fabricated with a structure of ITO/ZnO/ active layer/Al and hole‐only devices were fabricated with a structure of ITO/PEDOT:PSS/ active layer/MoO_x_/Ag. Mobility was calculated using Mott–Gurney relationship.

### Simulation

For the numerical simulations, an electro‐optical device model was used.^[^ ^20^ ^]^ The electrical behavior of the device was described by a 1D drift‐diffusion model, accounting for the injection, extraction, recombination, and space charge of electrons and holes in the active layer. The active bulk‐heterojunction layer was treated as an effective semiconductor with an energy level gap determined by the electron transport level of the acceptor and the hole transport level of the donor. The ITO/ZnO cathode and MoOx/Ag anode contact was assumed to be situated at *x* = 0 and *x* = *d*, respectively, where *x* denotes the position within the active layer and *d* the thickness of active layer; the contacts were assumed to be non‐selective with injection barriers of 0.1 eV for majority carriers. An undoped, trap‐free active layer dominated by bimolecular recombination was assumed.^[^ ^20^ ^]^ To obtain realistic charge carrier generation rates inside the active layer, a transfer‐matrix model was used to account for the wavelength‐dependent absorption and optical interference of light within the device stack.^[^ ^54^, ^55^ ^]^ A device structure of glass/ITO/ZnO/active layer/MoOx/Ag is considered, where the glass substrate was assumed to be incoherent.^[^ ^55^ ^]^ Subsequently, for a given incident light intensity, the absorption rate in the active layer was calculated using the refractive indices, extinction coefficients, and thicknesses of each layer as input.^[^ ^56^ ^]^ As light source either a 1000 lux white indoor LED or the AM1.5G spectrum was used as the illuminant.

## Conflict of Interest

The authors declare no conflict of interest.

## Supporting information



Supporting Information

Supporting Information

## Data Availability

The data that support the findings of this study are available from the corresponding author upon reasonable request.
