# Supplementary material for: High Thickness Tolerance in All‐Polymer‐Based Organic Photovoltaics Enables Efficient and Stable In‐Door Operation
Source: Adv Sci (Weinh). 2024 Sep 20;11(42):2408181. doi: 10.1002/advs.202408181 (PMC11558079; doi:10.1002/advs.202408181)
Supplement: Supplementary file 1 — Supporting Information [file ADVS-11-2408181-s001.pdf]

ADVANCED SCIENCE
Open Access

Supporting Information

for *Adv. Sci.*, DOI 10.1002/advs.202408181

High Thickness Tolerance in All-Polymer-Based Organic Photovoltaics Enables Efficient and Stable In-Door Operation

Lei Zhang, Seonjeong Lee, Song Yi Park, Oskar J. Sandberg, Emily J. Yang, Paul Meredith, Yun-Hi Kim and Ji-Seon Kim**

Supporting Information

High Thickness Tolerance in All-Polymer-Based Organic Photovoltaics Enables Stable and Efficient In-door Operation

Lei Zhang, Seonjeong Lee, Song Yi Park, Oskar J. Sandberg, Emily J. Yang, Paul Meredith, Yun-Hi Kim and Ji-Seon Kim**

L. Zhang, Dr. S. Y. Park[+], Emily J. Yang, Prof. J.-S. Kim
Department of Physics and Centre for Processable Electronics, Imperial College London
Prince Consort Road, London SW7 2AZ, UK
E-mail: ji-seon.kim@imperial.ac.uk

Dr. O. J. Sandberg
Physics, Faculty of Science and Engineering, Åbo Akademi University, Henrikinkatu 2, 20500 Turku, Finland

Prof. P. Meredith
Sustainable Advanced Materials (Sêr SAM) Group, Centre for Integrative Semiconductor Materials and Department of Physics, Swansea University, Singleton Park, Swansea SA2 8PP, UK

S. Lee, Prof. Y. H. Kim
Department of Chemistry and Research Institute of Molecular Alchemy (RIMA), Gyeongsang National University, Jinju, Gyeongnam 660-701, South Korea
E-mail: ykim@gnu.ac.kr

[+]Present address: Department of Physics, Pukyong National University, Busan 48513, Republic of Korea

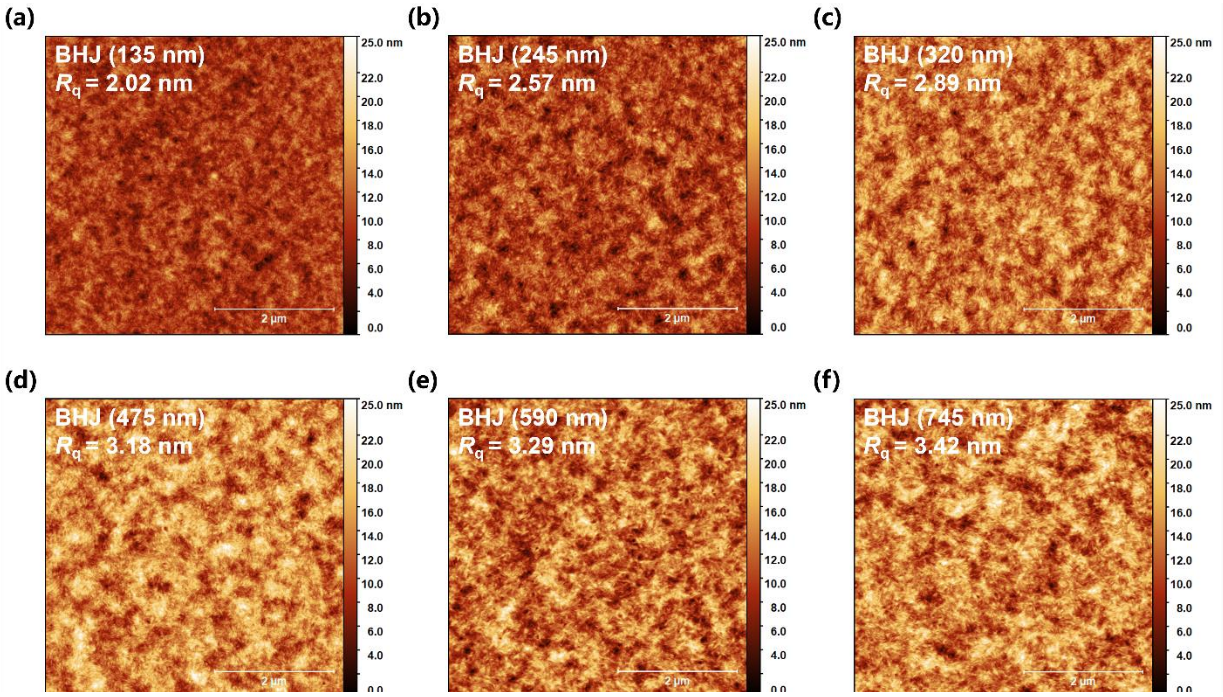

Figure S1. AFM height images of various-thickness BHJ films.

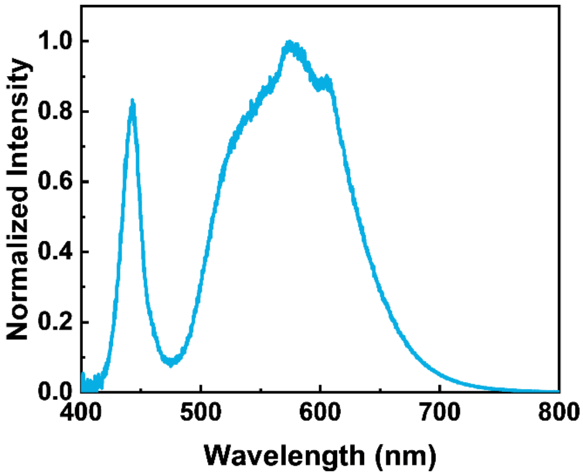

Figure S2. Emission spectra of white LED.

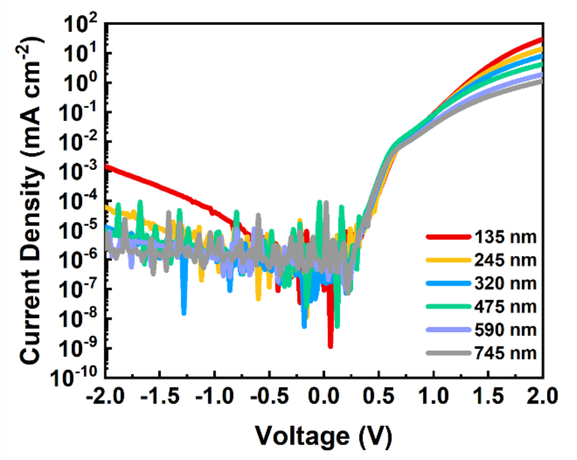

Figure S3. Dark *J*-*V* curves of various-thickness devices

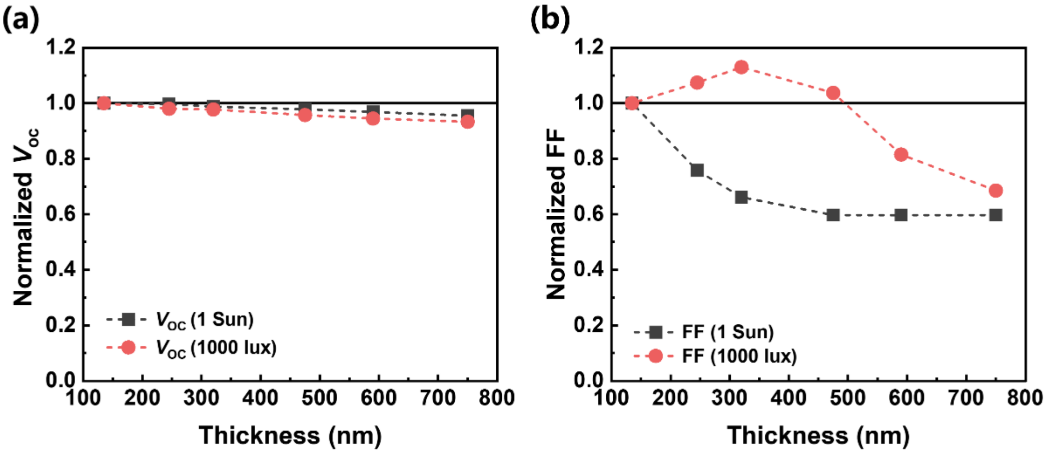

Figure S4. Normalized (a) V_{OC} and (b) FF as a function of thickness measured under 1 Sun and 1000 lux conditions.

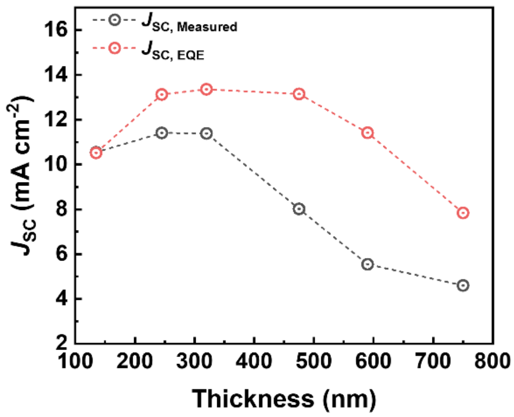

Figure S5. Comparison between measured J_{SC} ($J_{SC,\ Measured}$) and calculated J_{SC} from EQE spectra ($J_{SC,\ EQE}$) of various-thickness devices.

Table S1 Summary of calculated J_{SC} from EQE spectra ($J_{SC,\ EQE}$) of various-thickness devices.

	$J_{SC,\ EQE}$ [mA/cm^2]
135 nm	10.52
245 nm	13.13
320 nm	13.36
475 nm	13.15
590 nm	11.42
745 nm	7.84

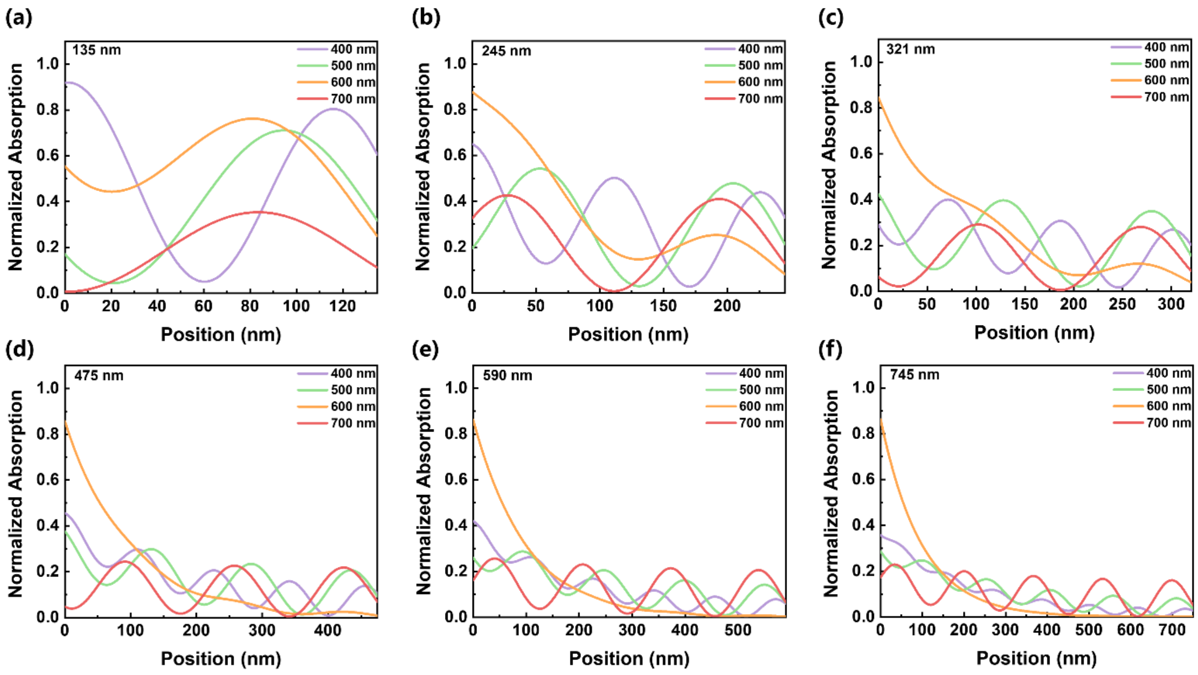

Figure S6. Normalized absorption profiles of (a) 135 nm, (b) 245 nm, (c) 320 nm, (d) 475 nm, (e) 590 nm, (f) 745 nm devices, as a function of the position inside the active layer at different photon wavelengths.

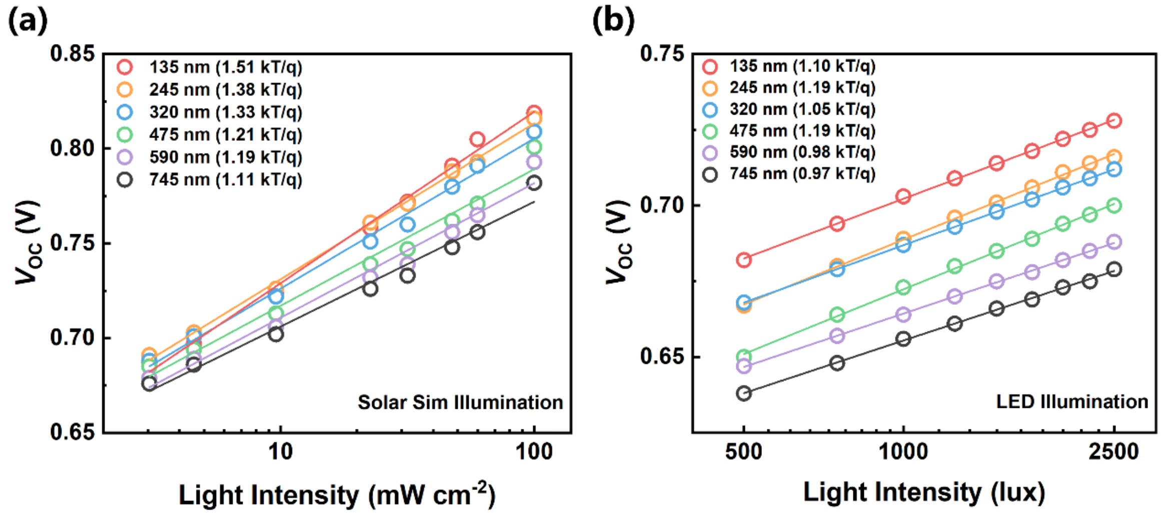

Figure S7. Light-intensity dependence of V_{OC} measuring using (c) solar simulator and (d) indoor light (1000 lux).

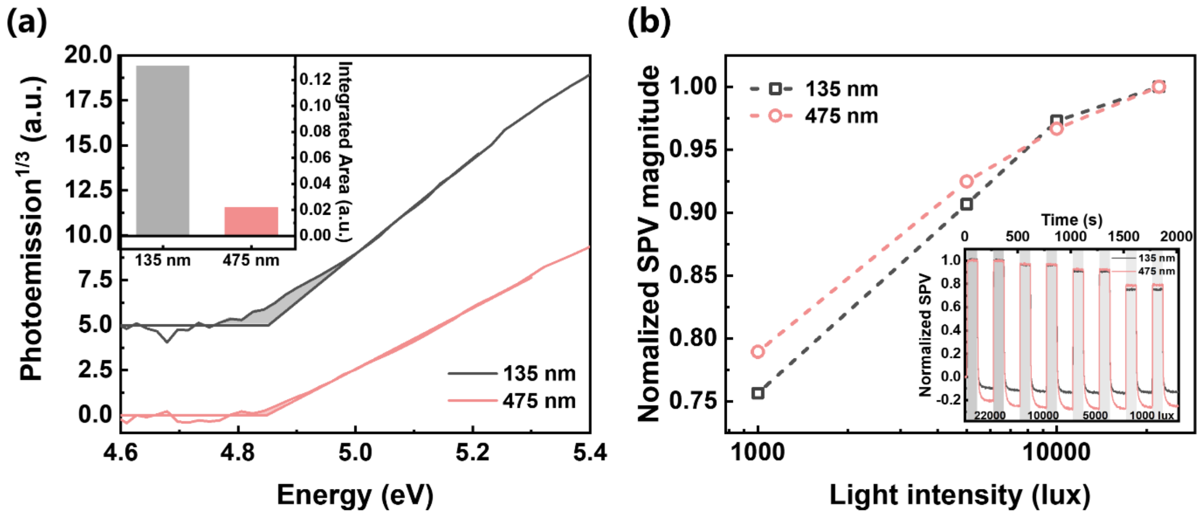

Figure S8. (a) Ambient photoemission (APS) spectra of blend films. The insert shows the integrated area extracted from APS spectra, indicating shallow trap density. (b) Normalized SPV magnitude for each intensity. The insert shows the normalized SPV measurements under different light intensities.

Table S2 Summary of hole and electron mobilities of neat and BHJ films.

	μ_h [$\times10^{-4}$ cm^2 V^{-1} s^{-1}]	μ_e [$\times10^{-5}$ cm^2 V^{-1} s^{-1}]	μ_h/μ_e
Neat PBDB-T	7.58	-	-
Neat Se-Th 0.8	-	7.78	-
BHJ (135 nm)	6.86	4.54	15
BHJ (245 nm)	5.13	1.96	26
BHJ (320 nm)	3.77	1.00	38
BHJ (475 nm)	2.86	0.53	54
BHJ (590 nm)	2.17	0.28	78
BHJ (745 nm)	1.89	0.21	90

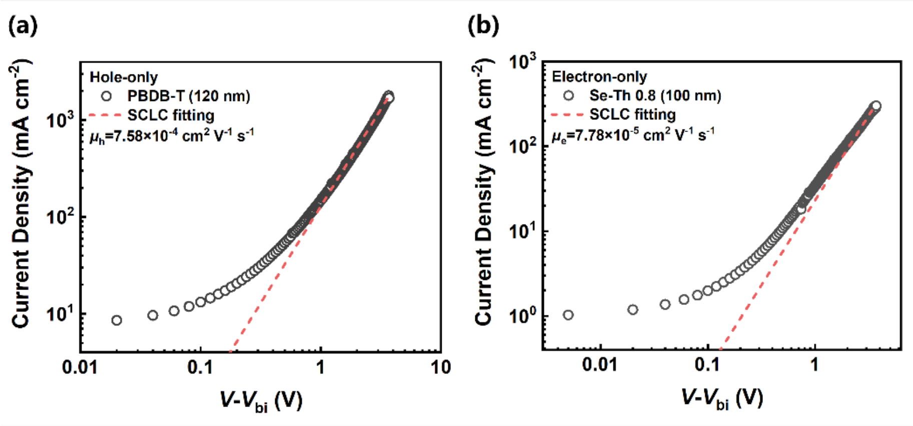

Figure S9. *J*-*V* characteristics of (a) hole-only and (b) electron-only devices with neat PBDB-T and Se-Th 0.8.

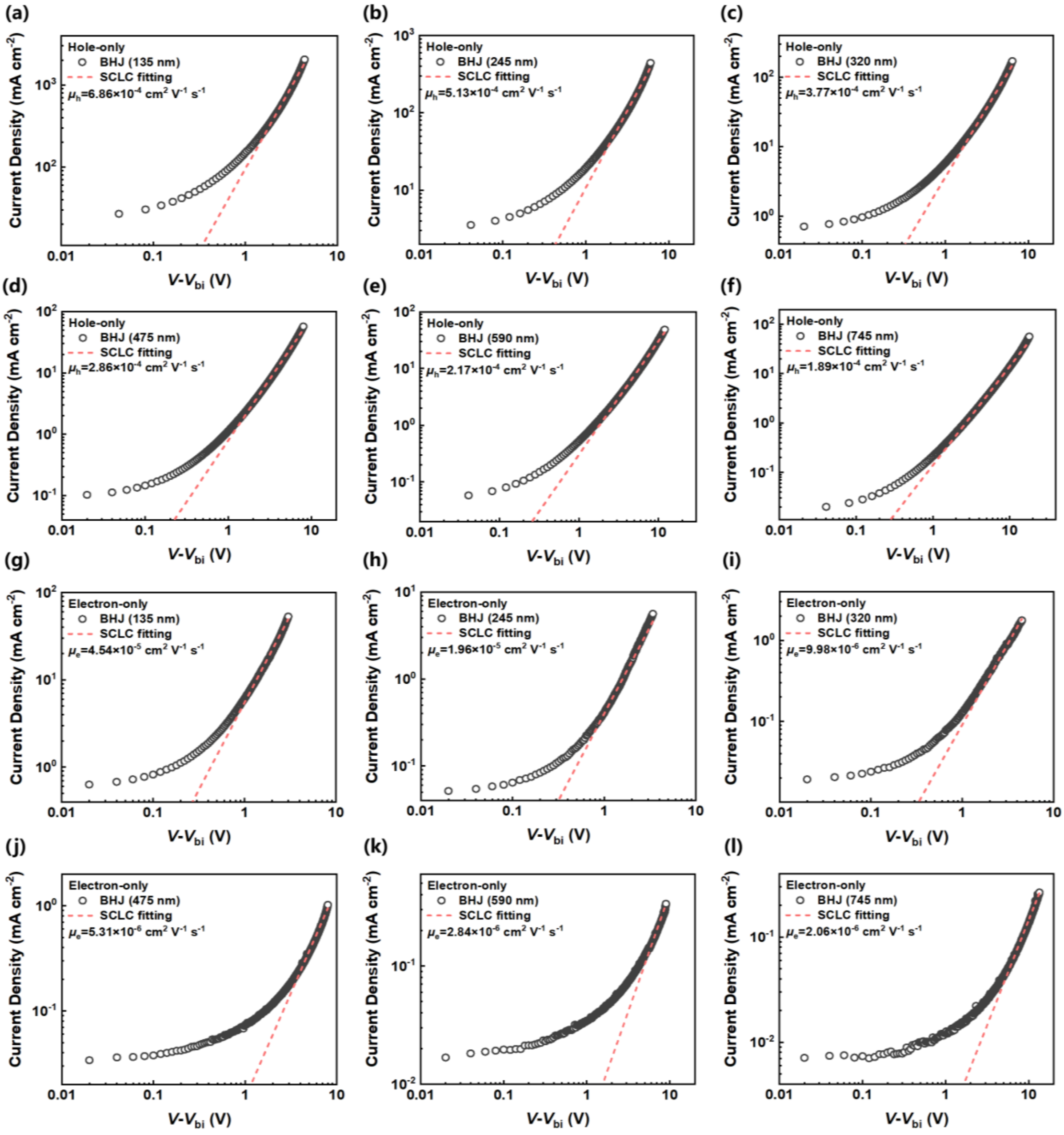

Figure S10. J-V characteristics of (a-f) hole-only and (g-l) electron only devices with various photoactive layer thicknesses.

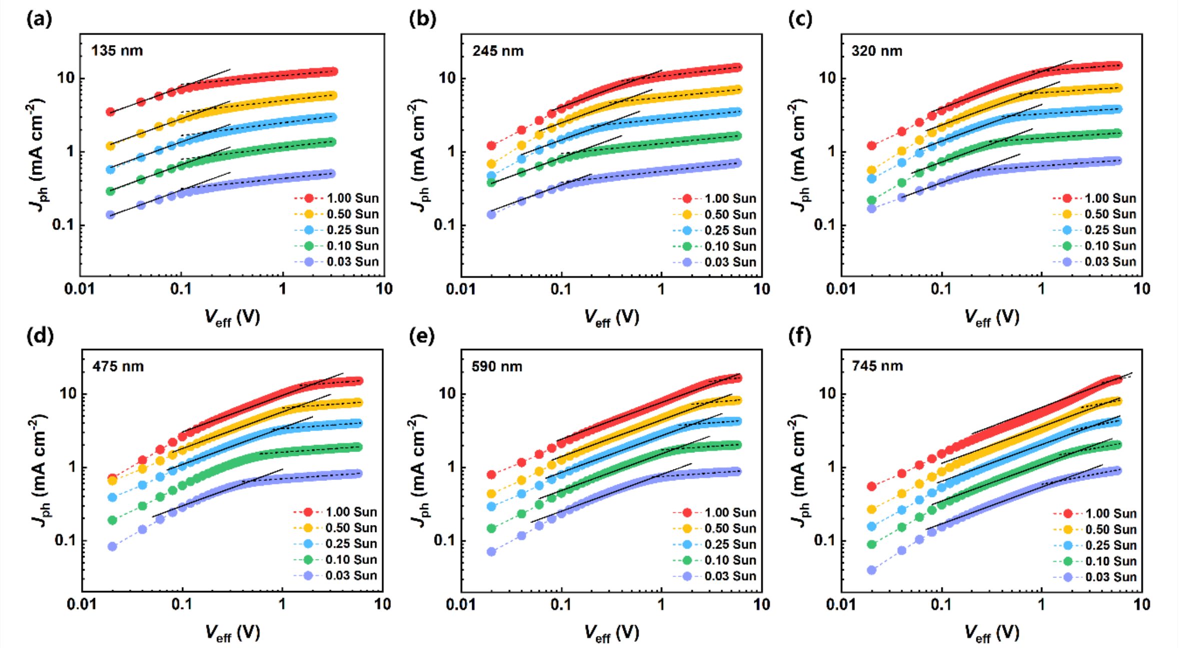

Figure S11. J_{ph} versus V_{eff} characteristics of (a) 135 nm, (b) 245 nm, (c) 320 nm, (d) 475 nm, (e) 590 nm, (f) 745 nm devices, measured using solar simulator with various light intensities.

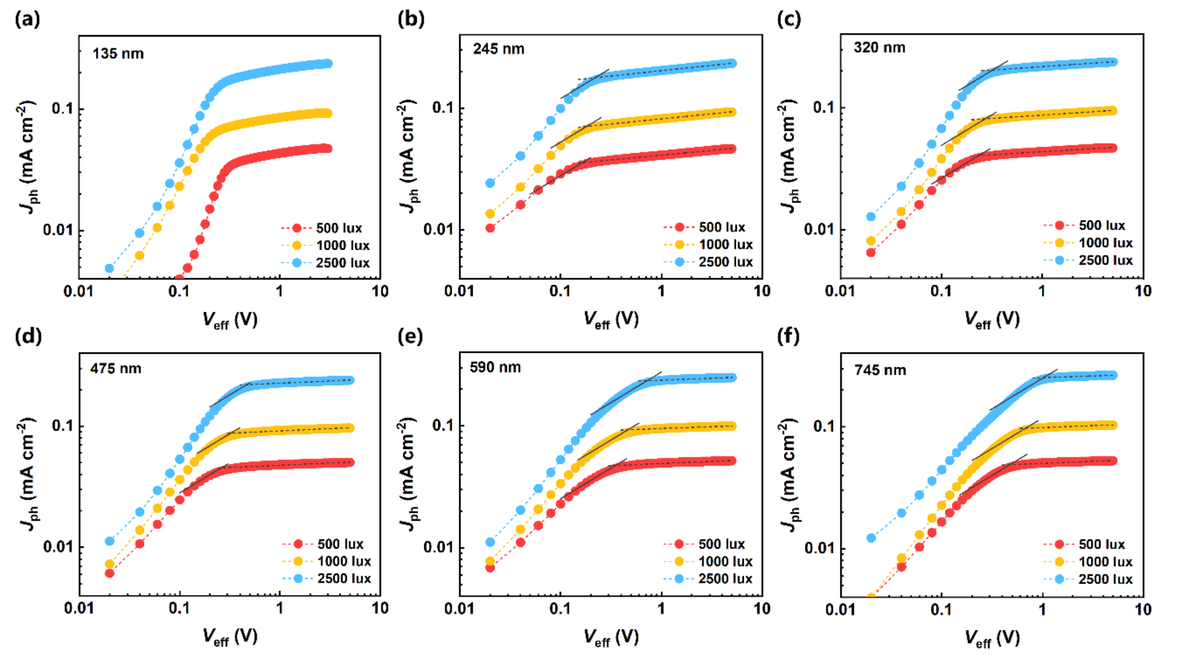

Figure S12. J_{ph} versus V_{eff} characteristics of (a) 135 nm, (b) 245 nm, (c) 320 nm, (d) 475 nm, (e) 590 nm, (f) 745 nm devices, measured using indoor lights with various light intensities.

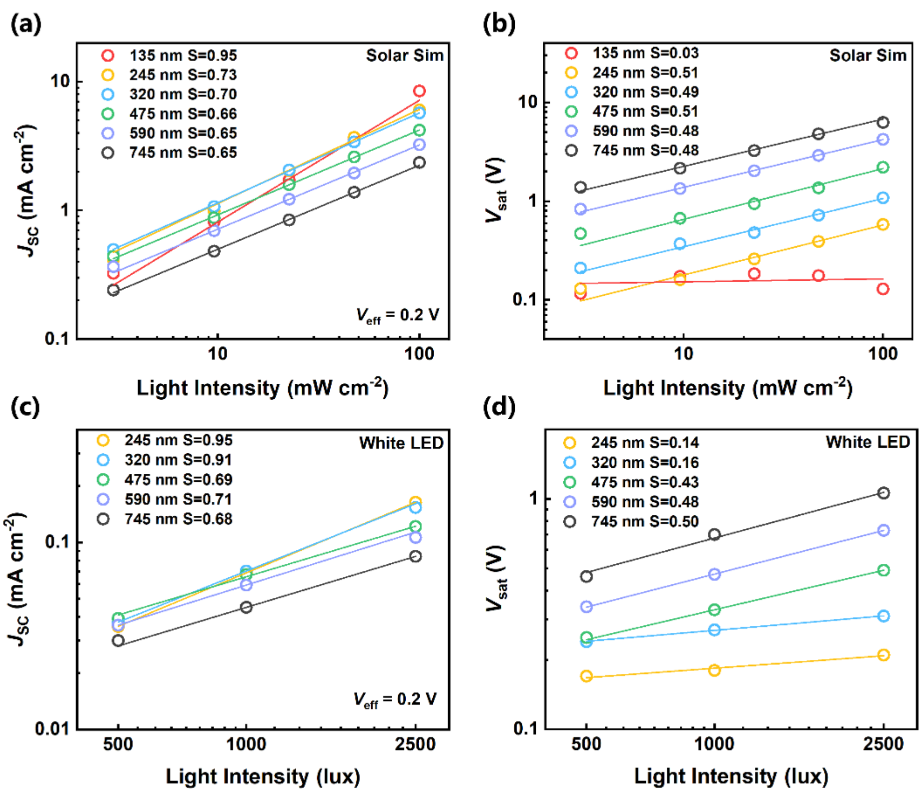

Figure S13. Light-intensity dependence of J_{ph} at V_{eff} = 0.2 V taken from (a) Figure S11 and (c) Figure S12. Light-intensity dependence of V_{sat} extracted from (b) Figure S10 and (d) Figure S11.

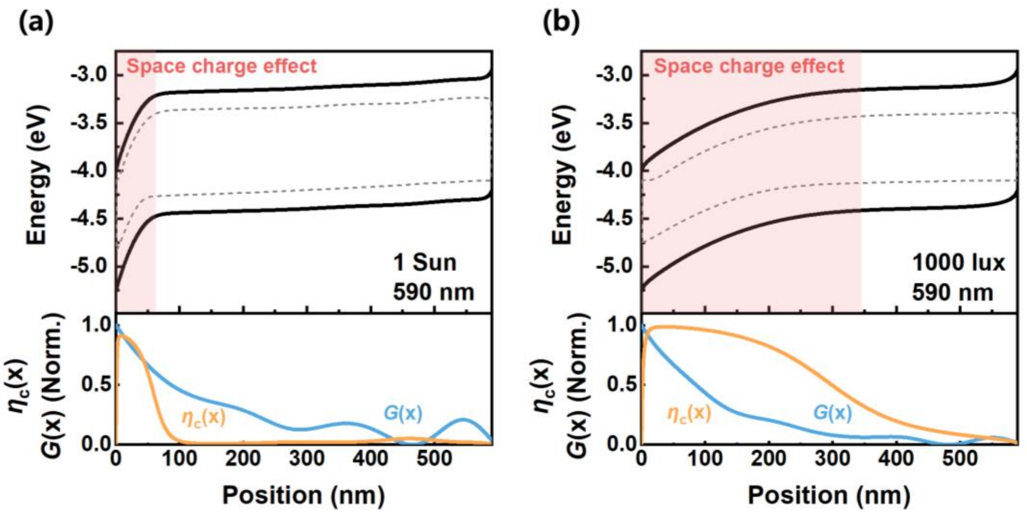

Figure S14. Simulated energy-level diagrams and corresponding collection efficiency (η_c(x)) and normalized generation rate (G(x)) plots of the 590-nm device under (a) 1 Sun and (b) 1000 lux conditions at short circuit.

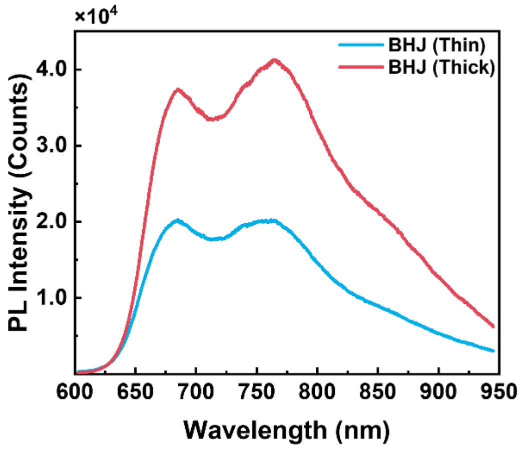

Figure S15. PL spectra of thin (135 nm) and thick (475 nm) blend films.

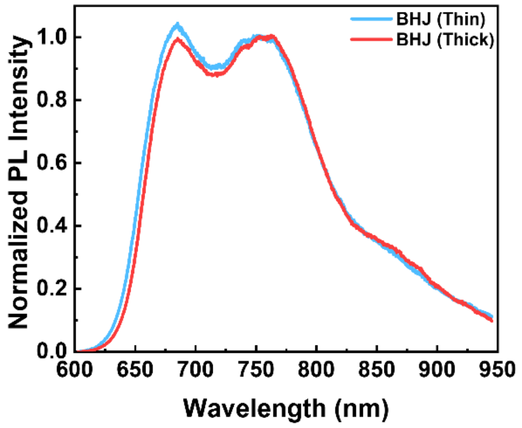

Figure S16. Normalized PL spectra of thin and thick blend films after subtraction of acceptor emission. Donor aggregation (indicated by a decrease in 685 nm donor peak) in thick films is further confirmed by subtracting the acceptor contribution in the blend PL spectra.

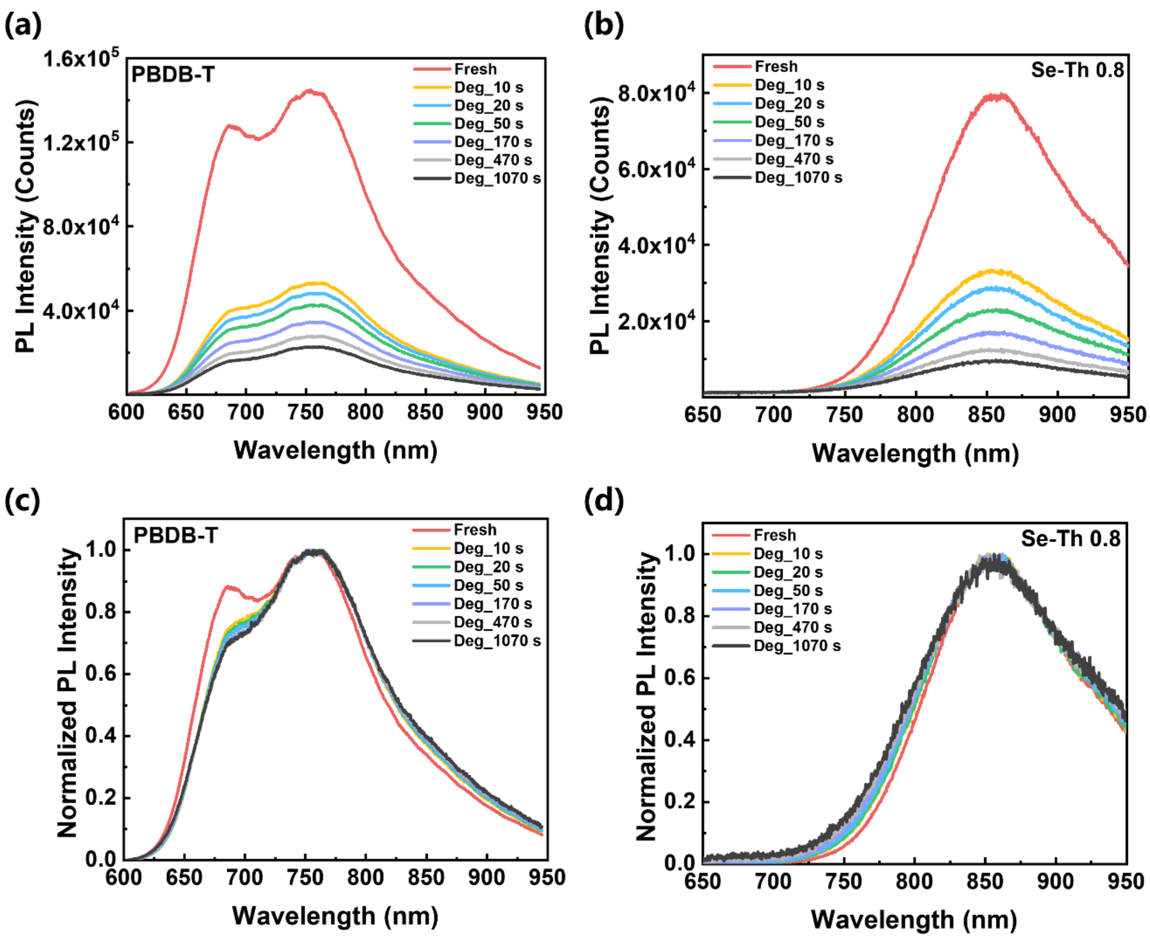

Figure S17. In-situ PL and the corresponding normalized spectra of the neat (a, c) PBDB-T and (b, d) neat Se-Th 0.8 films, taken at varied degradation times with a 514 nm laser.

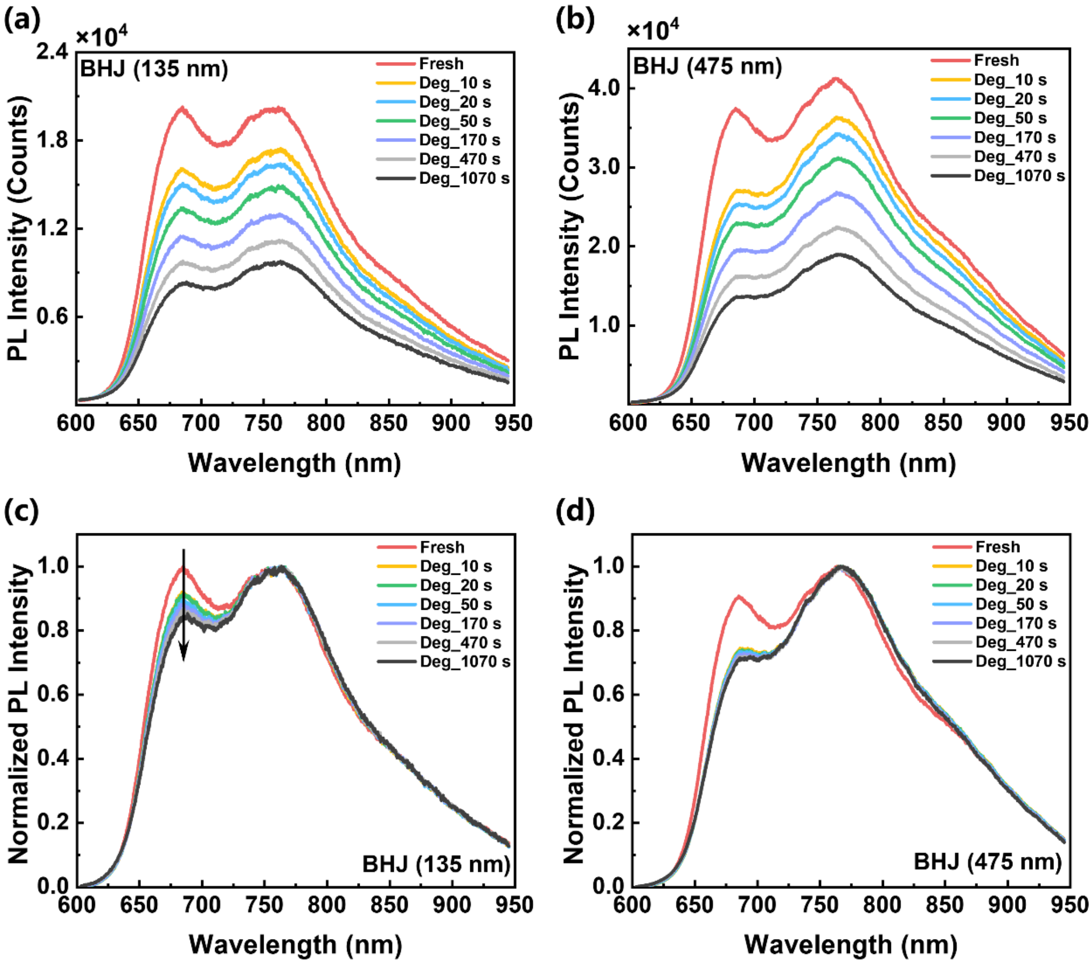

Figure S18. In-situ PL and the corresponding normalized spectra of the neat (a, c) thin (135 nm) and (b, d) thick (475 nm) films, taken at varied degradation times with a 514 nm laser. An initial rapid change in PL at 685 nm is observed. In the long-term stability data shown in Figure 6a and 6b, this rapid decay is not observed because the testing was conducted under 1000 lux, which has much lower light intensity than the PL testing conditions.

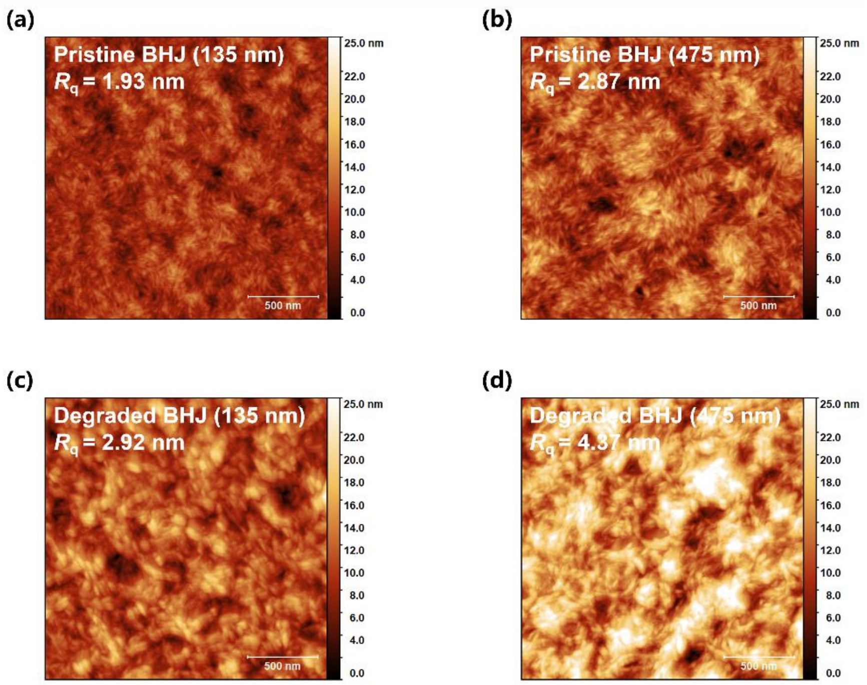

Figure S19. AFM height images of pristine and degraded of (a, c) 135-nm and (b, d) 476-nm devices. Films are degraded after continuous illumination under 1000 lux LED for 500 h.
